# Association of Rapid Weight Gain During Early Childhood With Cardiovascular Risk Factors in Japanese Adolescents

**DOI:** 10.2188/jea.JE20120107

**Published:** 2013-03-05

**Authors:** Yuki Fujita, Katsuyasu Kouda, Harunobu Nakamura, Masayuki Iki

**Affiliations:** 1 Department of Public Health, Kinki University Faculty of Medicine, Osaka-Sayama, Osaka, Japan; 1 近畿大学医学部公衆衛生学教室; 2 Department of Health Promotion and Education, Graduate School of Human Development and Environment, Kobe University, Kobe, Japan; 2 神戸大学大学院人間発達環境学研究科

**Keywords:** blood pressure, body weight change, infant, lipoproteins, obesity

## Abstract

**Background:**

Because of the lack of relevant data, we investigated the association between rapid weight gain (RWG) during early childhood and cardiovascular risk factors in Japanese adolescents.

**Methods:**

The source population comprised 2285 adolescents aged 13 to 14 years enrolled in any public school in Fukuroi City, Japan during 2008, 2009, or 2010. Since there are no private schools in this city, almost all adolescents who lived in the city went to 1 of these schools. We obtained data on blood pressure (BP), serum lipids, and anthropometry during adolescence, as well as anthropometry at birth, age 1.5 years, and age 3 years, from the Maternal and Child Health Handbook for 1624 children. RWG was defined as a change in body-weight standard deviation score greater than 0.67 from age 0 to 1.5 years or from age 1.5 to 3 years.

**Results:**

After adjusting for confounding factors, adolescents who had RWG from 0 to age 1.5 years or from age 1.5 to 3 years were more likely to be overweight. Adolescents who had RWG during both periods were more likely to be overweight (odds ratio [OR], 6.37; 95% CI, 3.06–13.24), have unfavorable lipid concentrations (OR, 2.03; 95% CI, 1.15–3.58), and have high BP (OR: 2.36, 95% CI: 1.34–4.13). The associations with unfavorable lipid concentrations and high BP disappeared after further adjusting for current body mass index.

**Conclusions:**

RWG during early childhood predicts unfavorable lipid concentrations and high BP in Japanese adolescents, and this relationship is mediated by body mass index in later life.

## Abstract (ja)

**【背景】:**

乳幼児期の体重増加とその後の心血管疾患危険因子との関連についての研究はほとんどない。今回は青年期の心血管疾患危険因子と乳幼児期の急速な体重増加（RWG）との関連について調査した。

**【方法】:**

標的集団は2008年、2009年あるいは2010年に袋井市の公立学校に在籍した2285名の生徒（中学2年生）であった。そのうち1624名から中学2年時の血圧値、血清脂質値、身体測定値のデータを入手し、乳幼児期の身体測定値は母子健康手帳から入手した。RWGは、出生時から1.5歳あるいは1.5歳から3歳の間の体重SDスコア変化が0.67以上として定義した。

**【結果】:**

交絡因子を調整後、出生時から1.5歳あるいは1.5歳から3歳の間にRWGがあった者は、RWGがなかった者と比べて、より過体重になる可能性が高かった。出生時から1.5歳と1.5歳から3歳の両期間にRWGがあった者も、より過体重になる可能性が高く（オッズ比, 6.37; 95% 信頼区間, 3.06-13.24）、さらに、好ましくない血清脂質値（オッズ比, 2.03; 95% 信頼区間, 1.15-3.58）や高い血圧値（オッズ比, 2.36; 95% 信頼区間, 1.34-4.13）を持つ可能性が高かった。この解析モデルにおいて、さらに現在のbody mass indexで調整した場合、RWGと好ましくない血清脂質値や高い血圧値との関連はなくなった。

**【結論】:**

乳幼児期のRWGは青年期の好ましくない血清脂質値や高い血圧値を予測する。この関連はその後の体格によって介在されている。

## INTRODUCTION

Unfavorable levels of serum lipoprotein cholesterol have long been recognized as a risk factor for coronary artery disease in adults.^1^ Studies have tracked serum lipid and lipoprotein levels from childhood through young adulthood,^2^^–^^4^ and 1 such study showed that cholesterol measurements obtained in childhood predicted adult levels of total cholesterol and low-density lipoprotein cholesterol (LDL-C).^4^ Hypertension and high blood pressure (BP) are also powerful risk factors for cardiovascular disease in adults.^5^^,^^6^ Strong evidence can be obtained by tracking BP from childhood to adulthood^7^: the Cardiovascular Risk in Young Finns Study followed participants for 27 years and showed that childhood BP correlated with values measured in middle age.^8^ Hypertension, high BP, and adverse levels of serum lipoprotein cholesterol in children have been identified as potent risk factors for future cardiovascular illness.^9^

Previous studies reported that rapid weight gain (RWG) during infancy was associated with low serum high-density lipoprotein cholesterol (HDL-C) and high BP at age 17 years,^10^ and that weight gain in the first 3 months of life was inversely associated with serum HDL-C in adults aged 18 to 24.^11^ A cohort study also reported that weight gain from birth to 3 months was positively associated with lipid levels and systolic blood pressure (SBP) at age 17 years.^12^ However, little is known about the association between growth in early life and subsequent cardiovascular risk factors.

In Japan, Maternal and Child Health Services (MCH Services) are regulated by the Law of Maternal and Child Health. The Japanese Maternal and Child Health System requires examinations of all children at birth, 1.5 years (age 1.5), and 3 years (age 3),^13^ during which height and weight are measured.^14^ These health examinations may predict cardiovascular risk factors in late childhood. We examined the association between growth in early childhood and cardiovascular risk factors in adolescence, using records from these physical examinations.

## METHODS

### Study participants

The source population was all eighth graders (aged 13–14 years; 2285 students: 1169 boys and 1116 girls) registered in any public school in Fukuroi, Japan in 2008, 2009, or 2010. Because Fukuroi has no private junior high schools, almost all adolescents aged 13 to 14 years in the city were included. The Fukuroi Board of Education conducts health examinations of all children aged 13 to 14 years at each school from April through June. With regard to the source population, data from health examinations, including anthropometric measurements, serum lipid levels, and BP levels, were obtained for 2225 students (1132 boys and 1093 girls; 97% of source population). In addition, data on height (length) and weight at birth, age 1.5, and age 3 were obtained for 1624 students (817 boys and 807 girls; 71% of source population) and included in the present analysis. This study was approved by the Ethics Committee of Kinki University Faculty of Medicine and conducted in accordance with the ethical principles of the Declaration of Helsinki.

### Health examination and risk factor measurements

Height and body weight were measured in accordance with the Japanese School Health Law by licensed teachers who have a role in health education and health care (*Yogo* teachers). Height was measured to an accuracy of 0.1 cm and weight to 0.1 kg. Body mass index (BMI) was then calculated as weight divided by height squared (kg/m^2^).

Measurements of resting SBP and diastolic blood pressure (DBP) were taken by nurses and medical technologists from the Shizuoka Prefecture Preventive Medicine Association (*Shizuokaken Yoboigakukyokai*, Shizuoka, Japan) using an automated device (BP-103N or BP-103i II, Colin Corporation, Komaki, Japan). Proper cuff size was selected based on arm circumference.^15^ Measurements were taken in the seated position, with the right arm supported at the level of the heart. If the value obtained was greater than the resting cut-off point (ie, SBP 135 mm Hg or DBP 80 mm Hg), the measurement was repeated. If the second value was still above the cut-off point, a third measurement was obtained. If the third value was also above the cut-off point, the lowest of the 3 values was recorded and analyzed.

Blood tests were conducted at a laboratory associated with the Shizuoka Prefecture Preventive Medicine Association. HDL-C and LDL-C levels were determined using commercial assays (Cholestest N HDL and Cholestest LDL, respectively, Sekisui Medical Co. Ltd., Tokyo, Japan) performed by the same researcher. Intra- and inter-laboratory coefficients of variation were less than 4%.

### Anthropometry in infancy and early childhood

Body weight and length at birth, as well as body weight and height at ages 1.5 and 3, were transcribed to a questionnaire using information from the Maternal and Child Health Handbook (MCH Handbook) kept by the parents. The MCH Handbook system in Japan is designed to promote health for all children^16^ and has an almost 100% participation rate.^17^ Health examinations at ages 1.5 and 3 (including body weight and height) are required by MCH Services^13^ and are included in the MCH Handbook.^17^

### Definitions

A standard deviation score (SDS) independent of sex was calculated as [(measured body weight − reference mean body weight)/reference body weight SD], using Japanese growth charts for 1993 as the reference.^18^ Changes in body weight SDS between birth and age 1.5 and between age 1.5 and age 3 were calculated as [SDS at age 1.5 − SDS at birth] and [SDS at age 3 − SDS at age 1.5], respectively.

A change in body weight SDS greater than 0.67 was defined as RWG, as previously described.^19^^,^^20^ Early childhood growth patterns were classified as no RWG (no RWG from birth to age 1.5 or from age 1.5 to 3), RWG from birth to age 1.5 (RWG from birth to age 1.5 but not from 1.5 to 3), RWG from 1.5 to 3 (RWG from age 1.5 to 3 but not from birth to 1.5), or RWG from birth to 1.5 and from 1.5 to 3 (RWG from birth to age 1.5 and from age 1.5 to 3).

The cut-off point for overweight was defined as a BMI of 22.27 for boys and 22.98 for girls, in accordance with the International Obesity Task Force.^21^ High BP was defined as a value greater than or equal to the age- and sex-specific 90th percentile for either SBP or DBP, as recommended by the 1996 Task Force Report on High Blood Pressure in Children and Adolescents.^22^ Unfavorable serum lipid level was defined as an LDL-C value equal to or greater than the 90th percentile or an HDL-C value less than or equal to the 10th percentile.

### Statistical analysis

Statistical calculations were performed with SAS software for Windows, ver. 9.1 (SAS Institute Japan Ltd., Tokyo, Japan). The level of significance was set at *P* < 0.05. The unpaired *t*-test was used to evaluate differences in the means of continuous variables between groups, the Tukey–Kramer method was used to compare variables between growth patterns, and the chi-square test was used to compare prevalence. Logistic regression was used to assess the associations of early-childhood growth pattern with overweight, unfavorable lipid level, and high BP in adolescence. Current maternal BMI was used to adjust for nutritional status as a potential confounder of cardiovascular risk factors.^23^

## RESULTS

The physical characteristics and cardiovascular risk factors of study participants are shown in Table 1. Height and weight were significantly higher for boys than for girls at birth and ages 1.5, 3, and 13 to 14. There were also significant differences between adolescent boys and girls in cardiovascular risk factors, namely BMI, SBP, DBP, HDL-C, and LDL-C.

**Table 1.** Physical characteristics and cardiovascular risk factors of participants

	Total (*n* = 1624)	Boys (*n* = 817)	Girls (*n* = 807)
At birth			
Weight, kg	3.03 ± 0.43	3.08 ± 0.44	2.98 ± 0.41^a^
Length, cm	49.1 ± 2.5	49.4 ± 2.6	48.8 ± 2.3^a^
BMI, kg/m^2^	12.5 ± 1.5	12.6 ± 1.5	12.5 ± 1.4
At age 1.5 years			
Weight, kg	10.4 ± 1.3	10.7 ± 1.3	10.2 ± 1.1^a^
Height, cm	79.2 ± 3.9	79.8 ± 4.1	78.7 ± 3.5^a^
BMI, kg/m^2^	16.6 ± 1.4	16.8 ± 1.4	16.4 ± 1.3^a^
At age 3 years			
Weight, kg	13.7 ± 1.6	13.9 ± 1.6	13.4 ± 1.5^a^
Height, cm	92.3 ± 4.0	92.8 ± 4.2	91.9 ± 3.6^a^
BMI, kg/m^2^	16.0 ± 1.2	16.1 ± 1.1	15.9 ± 1.3^a^
At age 13–14 years			
Weight, kg	46.8 ± 8.0	47.6 ± 8.9	46.0 ± 6.9^a^
Height, cm	156.5 ± 7.1	158.5 ± 7.9	154.4 ± 5.3^a^
BMI, kg/m^2^	19.1 ± 2.5	18.8 ± 2.5	19.3 ± 2.5^a^
SBP, mm Hg	109.9 ± 10.2	110.8 ± 9.9	108.9 ± 10.3^a^
DBP, mm Hg	59.3 ± 6.8	58.7 ± 6.6	59.9 ± 7.0^a^
HDL-C, mmol/L	1.74 ± 0.34	1.71 ± 0.33	1.78 ± 0.34^a^
LDL-C, mmol/L	2.28 ± 0.54	2.18 ± 0.51	2.38 ± 0.56^a^
Overweight, %	7.8	8.8	6.7

BMI, body mass index; SBP, systolic blood pressure; DBP, diastolic blood pressure; HDL-C, high-density lipoprotein cholesterol; LDL-C, low-density lipoprotein cholesterol.

Values are listed as mean ± standard deviation or as a percentage.

^a^*P* < 0.01 vs boys.

The present study classified participants based on infant growth patterns. About 32% of participants were in the birth to age 1.5 RWG group, 8% were in the age 1.5 to 3 RWG group, and 4% were in the age 0 to 1.5 and 1.5 to 3 RWG group. The anthropometric characteristics of participants in each group are shown in Table 2. As compared with the no RWG group, average birth weight was significantly lower and body weight at age 1.5 was significantly higher for the birth to age 1.5 RWG group. Birth weight for the age 0 to 1.5 and 1.5 to 3 RWG group was also significantly lower than in the no RWG group, while body weight at age 3 was significantly higher. As compared with the no RWG group, body weight was significantly lower at age 1.5 and significantly higher at age 3 in the age 1.5 to 3 RWG group.

**Table 2.** Early-childhood anthropometric characteristics of participants aged 13–14 years

	Growth pattern			

	No RWG (*n* = 887)	Only 0–1.5 RWG (*n* = 525)	Only 1.5–3 RWG (*n* = 137)	Both 0–1.5 & 1.5–3 RWG (*n* = 75)
At birth				
Weight, kg	3.18 ± 0.01	2.79 ± 0.02^a^	3.11 ± 0.03	2.80 ± 0.04^a^
Length, cm	49.6 ± 0.08	48.3 ± 0.10^a^	49.3 ± 0.20	48.6 ± 0.27^a^
BMI, kg/m^2^	13.0 ± 0.05	11.9 ± 0.06^a^	12.8 ± 0.12	11.7 ± 0.16^a^
At age 1.5 years				
Weight, kg	10.2 ± 0.04	11.0 ± 0.05^a^	9.4 ± 0.09^a^	11.2 ± 0.13^a^
Height, cm	79.1 ± 0.12	80.1 ± 0.16^a^	75.9 ± 0.31^a^	80.9 ± 0.42^a^
BMI, kg/m^2^	16.3 ± 0.04	17.2 ± 0.06^a^	16.0 ± 0.11	17.2 ± 0.15^a^
At age 3 years				
Weight, kg	13.1 ± 0.05	14.1 ± 0.06^a^	14.2 ± 0.12^a^	16.0 ± 0.16^a^
Height, cm	91.6 ± 0.13	93.1 ± 0.17^a^	92.6 ± 0.33^b^	95.6 ± 0.44^a^
BMI, kg/m^2^	15.7 ± 0.04	16.2 ± 0.05^a^	16.5 ± 0.10^a^	17.5 ± 0.13^a^

RWG, rapid weight gain; Only 0–1.5 RWG, rapid weight gain from 0 to age 1.5 but not from 1.5 to age 3; Only 1.5–3 RWG, rapid weight gain from age 1.5 to age 3 but not from 0 to age 1.5; Both 0–1.5 & 1.5–3 RWG, rapid weight gain from 0 to age 1.5 and age 1.5 to age 3; BMI, body mass index.

Values are listed as mean ± standard error and are adjusted for sex.

The Tukey–Kramer method was used to estimate mean differences between growth patterns.

^a^*P* < 0.01 and ^b^*P* < 0.05 vs no RWG.

Table 3 shows the anthropometric variables and cardiovascular risk factors of participants at age 13 to 14 categorized by growth pattern. The birth to age 1.5 RWG group had significantly higher BMI and SBP, and significantly lower HDL-C, than the no RWG group. The age 1.5 to 3 RWG group had a significantly higher BMI than the no RWG group. The birth to age 1.5 and 1.5 to 3 RWG group had significantly higher BMI, SBP, and DBP, and significantly lower HDL-C, than the no RWG group.

**Table 3.** Cardiovascular risk factors of participants aged 13–14 years categorized by growth patterns

	Growth pattern			

	No RWG (*n* = 887)	Only 0–1.5 RWG (*n* = 525)	Only 1.5–3 RWG (*n* = 137)	Both 0–1.5 & 1.5–3 RWG (*n* = 75)
Weight, kg	45.1 ± 0.26	48.2 ± 0.33^a^	48.9 ± 0.65^a^	54.0 ± 0.89^a^
Height, cm	155.3 ± 0.22	157.6 ± 0.29^a^	157.3 ± 0.57^a^	159.7 ± 0.77^a^
BMI, kg/m^2^	18.6 ± 0.08	19.3 ± 0.11^a^	19.7 ± 0.21^a^	21.1 ± 0.28^a^
SBP, mm Hg	108.8 ± 0.34	110.6 ± 0.44^a^	110.6 ± 0.85	115.7 ± 1.16^a^
DBP, mm Hg	58.9 ± 0.23	59.7 ± 0.29	59.0 ± 0.58	61.5 ± 0.78^a^
HDL-C, mmol/L	1.77 ± 0.01	1.71 ± 0.01^a^	1.71 ± 0.03	1.65 ± 0.04^b^
LDL-C, mmol/L	2.30 ± 0.02	2.23 ± 0.02	2.29 ± 0.05	2.39 ± 0.06
Overweight, %^c^	4.3	10.3	13.1	21.3
High BP, %^c^	16.7	20.0	21.2	36.0
Unfavorable serum lipid level, %^d^	16.7	18.9	22.6	30.7

RWG, rapid weight gain; Only 0–1.5 RWG, rapid weight gain from 0 to age 1.5 but not from 1.5 to age 3; Only 1.5–3 RWG, rapid weight gain from age 1.5 to age 3 but not from 0 to age 1.5; Both 0–1.5 & 1.5–3 RWG, rapid weight gain from 0 to age 1.5 and age 1.5 to age 3; BMI, body mass index; SBP, systolic blood pressure; DBP, diastolic blood pressure; HDL-C, high-density lipoprotein cholesterol; LDL-C, low-density lipoprotein cholesterol; BP, blood pressure.

Values are listed as mean ± standard error, adjusted for sex, or as a percentage.

The Tukey–Kramer method was used to estimate mean differences between growth patterns.

The chi-square test was used to detect group differences between growth patterns.

^a^*P* < 0.01 and ^b^*P* < 0.05 vs No RWG.

^c^*P* < 0.01, ^d^*P* < 0.05.

The odds ratio (OR) for each cardiovascular risk factor at age 13 to 14 was calculated in relation to growth pattern and compared with the no RWG group (Table 4). The birth to age 1.5 RWG group had a significantly higher OR for overweight, after adjusting for sex, birth-weight SDS, and current maternal BMI. The age 1.5 to 3 RWG group also had a significantly higher OR for overweight, after adjusting for sex, birth-weight SDS, and current maternal BMI. The birth to age 1.5 and 1.5 to 3 RWG group had significantly higher ORs for overweight, unfavorable lipid levels, and high BP, after adjusting for sex, birth-weight SDS, and current maternal BMI. When BMI at age 13 to 14 was entered into the model for adjustment, ORs for unfavorable lipid levels and high BP became insignificant. The results did not change when the analysis excluded infants with low birth weight (<2.5 kg; Table 5).

**Table 4.** Odds ratios for cardiovascular risk factors of participants aged 13–14 years, by growth pattern

Growth pattern	OR^a^	95% CI^a^	OR^b^	95% CI^b^	OR^c^	95% CI^c^
Overweight						
No RWG	1.00		1.00			
Only 0–1.5 RWG	2.57	1.67–3.96	2.86	1.74–4.68		
Only 1.5–3 RWG	3.47	1.91–6.28	3.46	1.84–6.49		
Both 0–1.5 & 1.5–3 RWG	6.77	3.53–12.98	6.37	3.06–13.24		
Unfavorable serum lipid levels						
No RWG	1.00		1.00		1.00	
Only 0–1.5 RWG	1.16	0.88–1.54	1.05	0.75–1.45	0.83	0.59–1.16
Only 1.5–3 RWG	1.45	0.93–2.24	1.34	0.84–2.14	1.04	0.64–1.69
Both 0–1.5 & 1.5–3 RWG	2.11	1.25–3.57	2.03	1.15–3.58	1.19	0.64–2.19
High blood pressure						
No RWG	1.00		1.00		1.00	
Only 0–1.5 RWG	1.25	0.95–1.65	1.10	0.80–1.52	0.95	0.68–1.32
Only 1.5–3 RWG	1.34	0.86–2.10	1.35	0.85–2.16	1.14	0.71–1.84
Both 0–1.5 & 1.5–3 RWG	2.81	1.69–4.66	2.36	1.34–4.13	1.66	0.92–2.99

OR, odds ratio; RWG, rapid weight gain; Only 0–1.5 RWG, rapid weight gain from 0 to age 1.5 but not from 1.5 to age 3; Only 1.5–3 RWG, rapid weight gain from age 1.5 to age 3 but not from 0 to age 1.5; Both 0–1.5 & 1.5–3 RWG, rapid weight gain from 0 to age 1.5 and age 1.5 to age 3; SDS, standard deviation score; BMI, body mass index.

^a^Adjusted for sex.

^b^Adjusted for sex, birth weight SDS, and maternal BMI.

^c^Adjusted for sex, birth weight SDS, maternal BMI, and BMI at age 13–14.

**Table 5.** Odds ratios for cardiovascular risk factors of participants aged 13–14 years, by growth pattern (excluding 144 participants with low birth weight)

Growth pattern	OR^a^	95% CI^a^	OR^b^	95% CI^b^	OR^c^	95% CI^c^
Overweight						
No RWG	1.00		1.00			
Only 0–1.5 RWG	2.73	1.75–4.27	2.58	1.61–4.12		
Only 1.5–3 RWG	3.25	1.75–6.03	3.05	1.59–5.84		
Both 0–1.5 & 1.5–3 RWG	7.14	3.51–14.52	6.00	2.78–12.95		
Unfavorable serum lipid levels						
No RWG	1.00		1.00		1.00	
Only 0–1.5 RWG	1.16	0.86–1.57	1.08	0.78–1.49	0.88	0.63–1.23
Only 1.5–3 RWG	1.50	0.96–2.35	1.40	0.87–2.26	1.10	0.67–1.82
Both 0–1.5 & 1.5–3 RWG	2.46	1.39–4.35	2.57	1.40–4.71	1.54	0.80–2.96
High blood pressure						
No RWG	1.00		1.00		1.00	
Only 0–1.5 RWG	1.28	0.95–1.72	1.20	0.87–1.64	1.06	0.77–1.47
Only 1.5–3 RWG	1.38	0.88–2.18	1.42	0.88–2.29	1.23	0.76–2.00
Both 0–1.5 & 1.5–3 RWG	2.76	1.57–4.86	2.52	1.36–4.65	1.82	0.95–3.46

OR, odds ratio; RWG, rapid weight gain; Only 0–1.5 RWG, rapid weight gain from 0 to age 1.5 but not from 1.5 to age 3; Only 1.5–3 RWG, rapid weight gain from age 1.5 to age 3 but not from 0 to age 1.5; Both 0–1.5 & 1.5–3 RWG, rapid weight gain from 0 to age 1.5 and age 1.5 to age 3; SDS, standard deviation score; BMI, body mass index.

^a^Adjusted for sex.

^b^Adjusted for sex and maternal BMI.

^c^Adjusted for sex, maternal BMI, and BMI at age 13–14.

## DISCUSSION

This study was the first to examine the association between early development and subsequent cardiovascular risk factors in Japanese children. Children who had RWG in early life were more likely to be overweight and have unfavorable lipid levels or high BP in later life. Postnatal growth patterns predicted cardiovascular risk factors in Japanese adolescents.

Only a few studies have reported an association between weight increase during early childhood and cardiovascular risk factors in later life.^10^^,^^11^^,^^24^^,^^25^ The Stockholm Weight and Pregnancy Development Study enrolled mostly whites and reported that weight increase at age 0 to 6 months was inversely associated with serum HDL-C, and positively associated with BP, at age 17 years.^10^ The Programming Factors for Growth and Metabolism Study in the Netherlands reported that weight increase in the first 3 months of life was inversely associated with serum HDL-C in early adulthood among a population composed almost entirely of whites.^11^ A southern Brazil population-based cohort study, which consisted primarily of participants of southern European descent, reported that weight increase from 20 to 42 months was associated with lower HDL-C and higher LDL-C in adolescence^24^ and that infant weight increase in the first 20 months was associated with higher SBP in adolescence.^25^ In the present Asian population, children who had RWG during early life were more likely to have unfavorable lipid levels or high BP in later life. Taken together, these findings suggest that postnatal growth is an important predictor of subsequent unfavorable lipid concentrations and high BP across ethnic groups.

As shown in Table 4, the association of RWG in early life with unfavorable lipid levels and high BP in later life, which was still significant after adjusting for potential confounding factors, disappeared when “BMI at age 13 to 14” was entered into the model for final adjustment. This suggests that RWG in early life is not related to unfavorable lipid levels or high BP in later life. However, we believe that BMI at age 13 to 14 is an intermediate factor (ie, intervening variable) between RWG in early life and unfavorable lipid levels and high BP in later life, rather than a confounding factor. Indeed, there is strong evidence for a relationship between BMI in early life and BMI in later life,^19^^,^^26^^–^^31^ and for a relationship between current BMI and current unfavorable lipid concentrations and high BP.^32^^–^^34^ Thus, BMI at age 13 to 14 should be considered an intervening variable and should thus not be used as an independent variable to analyze the relationship of RWG in early life with unfavorable lipid concentrations and high BP in later life.

A number of studies have reported that BMI in later life was an intermediate factor in unfavorable lipid concentrations and high BP in later life. A population-based birth cohort study reported that weight gain from age 2 to 4 years was related to an atherogenic lipid profile in adolescence and that this association was mediated by BMI in adolescence.^24^ A birth cohort study also reported that RWG in early childhood was related to BP in adults and that this relationship was mediated by adult BMI, which could be predicted by RWG in early childhood.^35^ The findings reported in these studies are consistent with our findings. However, an observational study reported an association between weight gain in the first 3 months of life and adult lipid levels, which remained significant after adjusting for adult body fat,^11^ although these findings may have resulted from sampling bias.

We used the MCH Handbook to collect data on early growth patterns. In Japan, health examinations of children at birth, 1.5 years, and 3 years are required by the Japanese Maternal and Child Health System.^13^ Height and weight are measured during this examination,^14^ and a record is included in the MCH Handbook. In a national survey, the mean body height (length) and weight of boys were 49.6 cm and 3.2 kg, respectively, at birth; 81.3 cm and 10.7 kg at age 1.5; and 93.3 cm and 13.8 kg at age 3. The mean body height and weight of girls were 48.9 cm and 3.1 kg, respectively, at birth; 79.6 cm and 10.1 kg at age 1.5 years; and 92.1 cm and 13.1 kg at age 3.^18^ Mean anthropometry values from the MCH Handbooks used in the present study were consistent with those in the national survey.

This study has some limitations worth noting. First, data were from only 1 city in Japan, and participants were thus not randomly selected from across the entire country. Therefore, sampling bias may be an issue. However, the mean body height and weight of the 2225 children who participated in the health examination conducted in the city (97% of source population) were similar to those in a national survey: 158.6 cm and 48.0 kg, respectively, for boys aged 13 to 14 years, and 154.2 cm and 46.3 kg, respectively, for girls aged 13 to 14 years.^36^ The HDL-C level in the 2225 children was 66 mg/dL in boys and 68 mg/dL in girls, whereas the Japanese nationwide averages were 61 mg/dL in boys and 63 mg/dL in girls.^37^ The LDL-C level in the 2225 children was 84 mg/dL in boys and 92 mg/dL in girls, whereas that measured in the Japanese nationwide study was 88 mg/dL for boys and 92 mg/dL for girls.^37^ Therefore, the present study population might be healthier than the Japanese national population.

Second, there may have been selection bias. However, mean body height, weight, lipid levels, and BP of children included in the present analysis (1624 children, 71% of source population) were similar to those of the 2225 children who participated in the health examination. However, the 1624 children with information from the MCH Handbook had significantly lower BMI (boys: 18.8 kg/m^2^ vs 19.2 kg/m^2^; girls: 19.3 kg/m^2^ vs 19.9 kg/m^2^, respectively) and higher HDL-C (boys: 1.71 mmol/L vs 1.66 mmol/L; girls: 1.78 mmol/L vs 1.72 mmol/L, respectively) as compared with children who lacked information from the MCH Handbook (601 students).

In conclusion, RWG from birth to age 1.5 or from age 1.5 to 3 was associated with overweight at age 13 to 14. RWG from birth to age 1.5 and from age 1.5 to 3 was associated with overweight, unfavorable lipid levels, and high BP at age 13 to 14. Rapid growth in early life predicts overweight, unfavorable lipid concentrations, and high BP in later life among Japanese children. The relationship of RWG in early childhood with unfavorable lipid concentrations and high BP in later life is mediated through BMI in later life.

## ONLINE ONLY MATERIALS

Abstract in Japanese.
