# Supplementary material for: Association of Rapid Weight Gain During Early Childhood With Cardiovascular Risk Factors in Japanese Adolescents
Source: J Epidemiol. 2013 Mar 5;23(2):103–8. doi: 10.2188/jea.JE20120107 (PMC3700244; doi:10.2188/jea.JE20120107)
Supplement: Abstract in Japanese. [file je-23-103-s001.pdf]

日本人の青年期における心血管疾患危険因子と乳幼児期の急速な体重増加の関連

藤田裕規[1]、甲田勝康[1]、中村晴信[2]、伊木雅之[1]

[1]近畿大学医学部公衆衛生学教室、[2]神戸大学大学院人間発達環境学研究科

【背景】乳幼児期の体重増加とその後の心血管疾患危険因子との関連についての研究はほとんどない。今回は青年期の心血管疾患危険因子と乳幼児期の急速な体重増加（RWG）との関連について調査した。

【方法】標的集団は 2008 年、2009 年あるいは 2010 年に袋井市の公立学校に在籍した 2285 名の生徒（中学 2 年生）であった。そのうち 1624 名から中学 2 年時の血圧値、血清脂質値、身体測定値のデータを入手し、乳幼児期の身体測定値は母子健康手帳から入手した。RWG は、出生時から 1.5 歳あるいは 1.5 歳から 3 歳の間の体重 SD スコア変化が 0.67 以上として定義した。

【結果】交絡因子を調整後、出生時から 1.5 歳あるいは 1.5 歳から 3 歳の間に RWG があった者は、RWG がなかった者と比べて、より過体重になる可能性が高かった。出生時から 1.5 歳と 1.5 歳から 3 歳の両期間に RWG があった者も、より過体重になる可能性が高く（オッズ比, 6.37; 95% 信頼区間, 3.06-13.24）、さらに、好ましくない血清脂質値（オッズ比, 2.03; 95% 信頼区間, 1.15-3.58）や高い血圧値（オッズ比, 2.36; 95% 信頼区間, 1.34-4.13）を持つ可能性が高かった。この解析モデルにおいて、さらに現在の body mass index で調整した場合、RWG と好ましくない血清脂質値や高い血圧値との関連はなくなった。

【結論】乳幼児期の RWG は青年期の好ましくない血清脂質値や高い血圧値を予測する。この関連はその後の体格によって介在されている。

キーワード：血圧、体重変化、乳幼児、青年期、リポタンパク質
